# Quantification of cell cycle re-entry during dedifferentiation of primary adipocytes *in vitro*

**DOI:** 10.1080/21623945.2024.2376571

**Published:** 2024-07-11

**Authors:** Ewa Bielczyk-Maczynska

**Affiliations:** a The Hormel Institute, University of Minnesota, Austin, MN, USA; b The Institute for Diabetes, Obesity, and Metabolism, University of Minnesota, Minneapolis, MN, USA; c Lillehei Heart Institute, University of Minnesota, Minneapolis, MN, USA; d Division of Cardiovascular Medicine, Department of Medicine, Stanford University, Stanford, CA, USA; e Department of Chemical and Systems Biology, Stanford University, Stanford, CA, USA

**Keywords:** Adipocyte, dedifferentiation, cell cycle, lineage tracing, DFAT cells

## Abstract

Dedifferentiated adipose tissue (DFAT) has been proposed as a promising source of patient-specific multipotent progenitor cells (MPPs). During induced dedifferentiation, adipocytes exhibit profound gene expression and cell morphology changes. However, dedifferentiation of post-mitotic cells is expected to enable proliferation, which is critical if enough MPPs are to be obtained. Here, lineage tracing was employed to quantify cell proliferation in mouse adipocytes subjected to a dedifferentiation-inducing protocol commonly used to obtain DFAT cells. No evidence of cell proliferation in adipocyte-derived cells was observed, in contrast to the robust proliferation of non-adipocyte cells present in adipose tissue. We conclude that proliferative MPPs derived using the ceiling culture method most likely arise from non-adipocyte cells in adipose tissue.

## Introduction

Stem cell-based therapies that combine tissue engineering and biomaterials have the potential to provide novel solutions for the treatment of a variety of diseases. Sources of patient-specific stem and progenitor cells for regenerative medicine include bone marrow-, adipose tissue- and umbilical cord-derived mesenchymal stem cells (MSCs) and induced pluripotent stem cells (iPSCs) [1]. Multipotent progenitor cells (MPPs) obtained through dedifferentiation of adipocytes (fat cells) have been suggested as an attractive alternative since the isolation of large quantities of adipose tissue is relatively non-invasive. In addition, adipocytes compose a much larger fraction of adipose tissue than MSCs (18.2% compared to 4.8% in human WAT, 19.8% compared to 5.3% in mouse WAT) [2], suggesting that dedifferentiated adipocytes (DFAT cells) could provide higher yield of stem cells than directly isolated adipose tissue-derived MPPs.

DFAT cells are obtained through the ceiling culture method, in which mature adipocytes adhere to the top surface of a tissue culture flask that is completely filled with media. After approximately 8 d, a large number of proliferating DFAT cells adhering to the tissue culture plate are detected. DFAT cells have fibroblast-like morphology and express markers of embryonic stem cells [3,4]. These cells can be further cultured for extended periods of time, or be differentiated into endothelial-like cells, osteocytes, chondrocytes or cardiomyocytes [4].

Due to the high cellular heterogeneity of adipose tissue, DFAT cells may be derived from contaminating non-adipocyte cells. In fact, differentiated cell types, such as adipocytes, have long been thought to have reached a permanent post-mitotic state. However, recent findings broadened our understanding of cellular plasticity and revealed that many differentiated cell types can resume proliferation under specific physiological and experimental conditions, including adipocytes present in the mammary gland [5] and skin [6]. It is not feasible to conduct lineage tracing experiments to validate the cellular origin of patient-derived DFAT cells. However, rodents have been successfully used to recapitulate key properties of human stem cell and adipocyte biology. Here, lineage tracing in a mouse model has been applied to test whether adipocytes give rise to proliferative cells obtained through two methods: (1) ceiling culture of adipocytes and (2) replating of primary adipocytes that were differentiated *ex vivo* using primary stromal vascular fraction (SVF) isolated from an ingunal fat pad. These findings support recent reports which found that although adipocytes undergo molecular changes associated with dedifferentiation *in vivo*, they do not show robust cell cycle re-entry or proliferation.

## Materials and methods

### Mice

All animal studies were conducted in accordance with the guidelines and regulations of the Administrative Panel on Laboratory Animal Care at the Stanford University School of Medicine. Mice were purchased from Jackson Laboratory. Homozygous mT/mG B6.129(Cg)-Gt(ROSA)26Sortm4(ACTB-tdTomato,-EGFP)Luo/J (cat. 007676) mice and nT/nG B6;129S6-Gt(ROSA)26Sortm1(CAG-tdTomato*,-EGFP*)Ees/J mice (cat. 023035) were bred to B6;FVB-Tg(Adipoq-cre)1Evdr/J mice (cat. 010803) and genotyped. In each biological replicate, adipose tissue from an individual mouse was isolated. Both male and female mice were used. Animals were euthanized using isofluorane euthanasia followed by cervical dislocation. All animal studies followed the ARRIVE guidelines 2.0 (https://arriveguidelines.org) [7].

### Cell isolation and culture

Primary adipocytes for ceiling culture and SVF cells for *ex vivo* differentiation were isolated from inguinal subcutaneous fat pads of 4–8-week-old female and male *Adipoq:Cre mT/mG* mice using a previously published approach [8]. Adipose tissue was minced and digested in a solution of collagenase type D (Roche 11,088,866,001, 1 mg/ml) and Dispase II (Sigma-Aldrich, D4693, 1 mg/ml) in phosphate-buffered saline with 1 mM CaCl_2_ for 40 min at 37°C with gentle shaking. Next, the solution was passed through sterile nylon mesh and centrifuged at 300 RCF for 5 min. The floating fraction and the pellet were separated to obtain adipocytes for ceiling culture and SVF for *ex vivo* differentiation, respectively.

To obtain SVF, the pellet was resuspended in culture medium (DMEM with 10% foetal bovine serum (FBS, Atlanta Biologicals) + 100 U/mL penicillin/streptomycin) with 2.5 mg/ml amphotericin B for 2–4 h. Next, cells received fresh culture medium and were grown in the presence of 2.5 mg/ml amphotericin B for up to 7 d before the start of the differentiation protocol. To differentiate the SVF, cells were plated in 12-well cell culture plates at 120,000 cells per well (day −1). At day 0, cells were treated with 250 mM 3-Isobutyl-1-methylxanthine (IBMX, Sigma-Aldrich), 1 mM dexamethasone (Sigma-Aldrich), 1.75 nM insulin (Sigma-Aldrich) and 500 nM rosiglitazone (Cayman Chemical) in culture medium. On day 2, cells were treated with 1.75 nM insulin and 500 nM rosiglitazone, and on day 4 with 1.75 nM insulin in a culture medium for two more days. Differentiated SVF was maintained in a culture medium with 1.75 nM insulin until day 6 when cells were trypsinized and replated at subconfluence in a 96-well format and subjected to EdU incorporation assay as described.

Ceiling culture was conducted through the introduction of the floating fraction obtained following the centrifugation of digested adipose tissue into a 15 mL tissue culture flask completely filled with culture medium. The flasks were then incubated upside down for 5 d, after which the culture media were replaced and the flasks were reversed. Cells were cultured for 7 more days prior to passaging at low density into 96-well plates and additional culture for an additional 18 d with culture media replaced every 3 d.

### 5-ethynyl-2’-deoxyuridine (EdU) incorporation assay

Living cells were exposed to 5-ethynyl-2’-deoxyuridine (EdU, Carbosynth), which was added to the culture media. For DFAT cells, EdU at the concentration of 1 mM was added for 30 min prior to fixation. For SVF-derived cells, EdU was added at the concentration of 0.5 μM 24 h prior to fixation. Samples were fixed in 4% paraformaldehyde/PBS for 30 min at room temperature. EdU was then visualized using Click-iT EdU Alexa Fluor 647 Imaging Kit (Thermo Fisher Scientific) according to the manufacturer’s instructions, followed by immunocytochemical staining of GFP as described.

### Immunocytochemical staining

Cells were fixed with 4% paraformaldehyde (PFA) in PBS for 30 min at room temperature, followed by three washes with PBS. Cells were then permeabilized with 0.1% Triton X-100 in PBS for 15 min on ice, followed by blocking with 5% bovine serum albumin (BSA, Sigma Aldrich) in PBS, and incubation with chicken anti-GFP (Fisher Scientific, NB1001614, 1:1,000) or anti-phH3 (Abcam, ab5176, 1:1,000) primary antibodies in 2% BSA in PBS overnight at 4°C. After washing, cells were incubated with Hoechst (1:10,000) and anti-chicken AlexaFluor488 or anti-rabbit AlexaFluor647 secondary antibodies (Thermo Fisher Scientific, 1:1,000). Cells were washed three times with PBS prior to imaging.

### Fluorescent imaging

The imaging of DFAT cells was conducted on a 3i (Nikon) epifluorescent microscope with a 20X objective. Automated fluorescent cell imaging was conducted using ImageXpress MicroXL (Molecular Devices, USA) epifluorescent microscope with a 10X objective and 1 × 1 binning. Cells were plated in optically clear 96-well plastic-bottom plates (Costar, #3904). Several non-overlapping sites in each well were imaged.

### Microscopy data processing

Microscopy images of DFAT cells were processed using ImageJ and scored manually using ImageJ version 1.54f. Automated processing of fluorescent images of SVF-derived cells was conducted in MATLAB R2016a (MathWorks). Data were obtained by automated image segmentation and measurement using the MACKtrack package [9], as previously described [10]. Individual nuclei were detected using the Hoechst signal in the DAPI channel, and GFP, RFP and Cy5 (EdU) signals were measured over nuclei. GFP and RFP signals used were averaged over the nucleus area, and the EdU signal was integrated over the nucleus. Cut-off values for GFP and EdU signals were manually selected and kept constant across all samples.

### Modeling of cell population composition

Excel v2402 (Microsoft) and GraphPad PRISM v. 7.04 were used to calculate and plot cell population growth. The following formulae were used to estimate the fraction of adipocyte and non-adipocyte cells:

adipocyte_*n*__+1_=adipocyte_*n*_ x survival

other_*n*__+1_=other_*n*_ x survival x (1+(24/division_time))

other_fraction_*n*_=other_*n*_/(other_*n*_+adipocyte_*n*_)*100

Equal survival of adipocytes and non-adipocytes was assumed, and it was fixed at 99% over every 24 h period. Data were calculated using Excel and plotted in PRISM and are provided as Supplementary Data.

### Quantification and statistical analysis

Unless specified otherwise, data are expressed as mean ± standard error of the mean (S.E.M). *p*-values <0.05 were considered statistically significant. Analyses were performed using PRISM software v. 7.04. Figures were prepared using Biorender.

## Results

**Figure 1.**
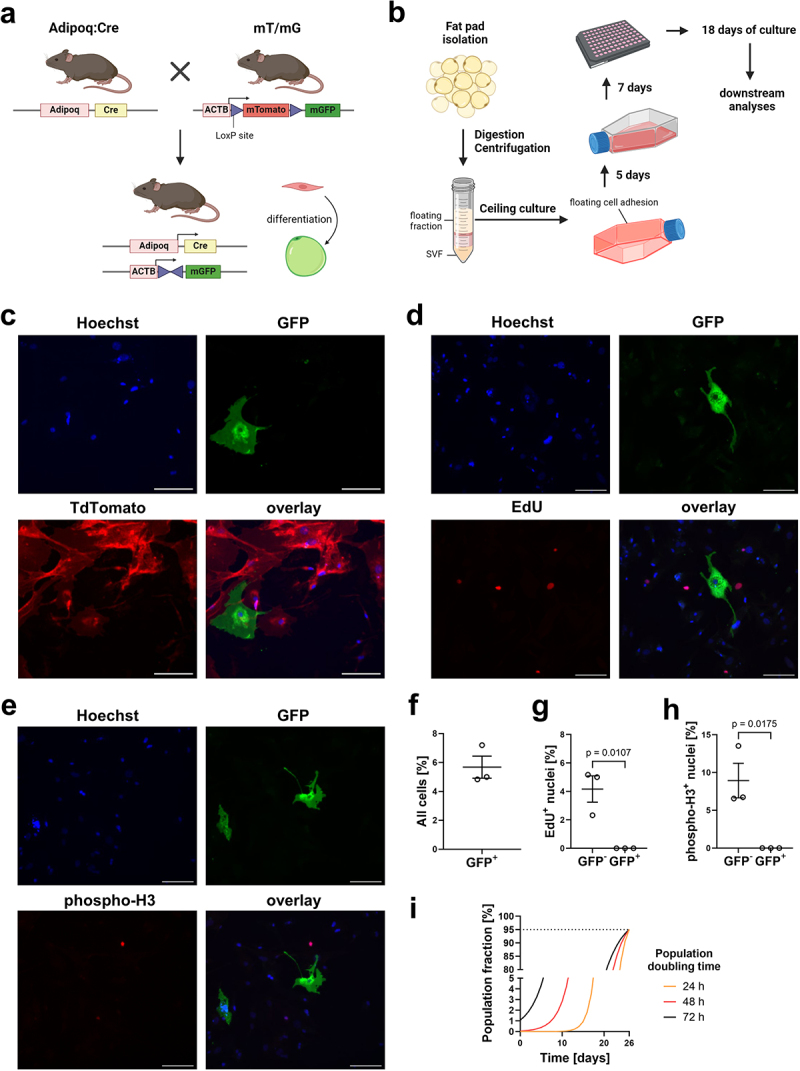
The majority of proliferative mouse cells identified using ceiling culture do not originate from adipocytes.

To determine whether the proliferative DFAT cells are in fact derived from mature adipocytes, the transgenic *Adipoq:Cre mT/mG* mouse model was used (Figure 1(a)). In these mice, an irreversible switch from red to green fluorescence occurs exclusively in the cell membrane of adipocytes [10,11]. Adipocyte-containing floating fraction was isolated from inguinal subcutaneous adipose tissue and subjected to ceiling culture for 12 d, followed by passaging of cells which adhered to the ceiling of the culture flask. Cells were cultured for additional 18 d prior to endpoint detection of cell proliferation using two complementary methods (Figure 1(b)). EdU incorporation assay, using a 30 min incubation of cells with EdU, was applied to detect DNA replication, indicative of cells in the S phase of the cell cycle [12], and the expression of phosphohistone H3 was used to detect cells in the late G2 and M phases of mitosis [13]. GFP^+^ adipocyte-derived cells on average constituted only 5.7% of all cells present at the end of the experiment (Figure 1(c,f)). Moreover, cell proliferation was detected only in GFP^−^ cells by both EdU incorporation and phospho-histone 3 (phospho-H3) immunohistochemistry (Figure 1(d,e,g,h)). Although all proliferative cells obtained through ceiling culture were derived from non-adipocytes, the adipocyte-derived cells displayed fibroblast-like morphology, indicative of morphological dedifferentiation (Figure 1(c–e)).

(a) The transgenic mouse model used for lineage tracing experiments. (b) Outline of the DFAT cell isolation using the ceiling culture method. (c) Representative fluorescent microscopy image of an adipocyte-derived GFP-positive cell (green) and TdTomato-positive-cells (red). Nuclei counterstained with Hoechst (blue). (d) Representative fluorescent images of DFAT cells subjected to the EdU incorporation assay. (e) Representative fluorescent images of DFAT cells subjected to immunofluorescent staining of the mitotic marker phospho-histone H3 (phospho-H3). (c−e) Scale bars: 100 μm. (f) Quantification of the fraction of GFP^+^ cell population. Total: 27/440 cells GFP^+^. (g) Quantification of EdU incorporation in GFP^+^ and GFP^−^ cells. EdU^+^GFP^−^, n = 8; EdU^−^GFP^−^, n = 196; EdU^+^GFP^+^, n = 0; EdU^−^GFP^+^, n = 13. (h) Quantification of phospho-H3 staining in GFP^+^ and GFP^−^ cells. phH3^+^GFP^−^, n = 21; phH3^−^GFP^−^, n = 218; phH3^+^GFP^+^, n = 0; phH3^−^GFP^+^, n = 23. (f-h) Average ± standard error of the mean shown. n = 3. Two-tailed Student *t* tests, *p*-value is indicated. (i) Estimation of the initial and final percentage of proliferating cells, when mixed with non-proliferating adipocytes, depending on the doubling time.

Given the low percentage of GFP^+^ cells present in the sample, we then estimated the initial frequency of proliferative cells needed to amount to the observed cell frequencies after 26 d of culture after cell isolation. Although it is dependent on the doubling time of proliferative cells present after isolation, even 1% of proliferative cells would be sufficient to give rise to over 95% of the cellular population after 26 d of culture, when mixed with non-proliferative cell population (Figure 1(i)).

**Figure 2.**
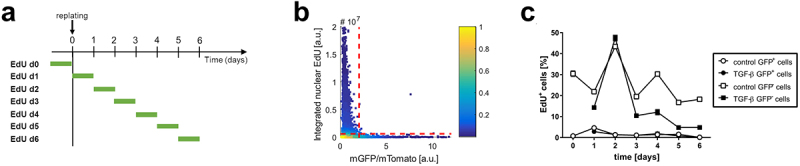
SVF-derived adipocytes do not re-enter cell cycle, irrespective of the presence of TGF-β.

The ceiling culture method does not allow for the analysis of early timepoints after cell purification, which could lead to the omission of early and transient cell cycle re-entry in adipocytes. To determine if adipocytes re-enter cell cycle shortly after being subjected to dedifferentiation-inducing stimuli, preadipocyte-containing SVF from *Adipoq:Cre nT/nG* mice, in which the fluorescent switch from tdTomato to GFP occurs in the nuclei of adipocytes [10], was differentiated *ex vivo* to obtain a mixture of differentiated GFP^+^ adipocytes and other GFP^−^ cell populations. The cells were then subjected to replating at non-confluent density to induce cell cycle re-entry. In addition, transforming growth factor β (TGF-β), whose role as a factor stimulating DFAT cell derivation was previously reported [14], was added to the culture media. To quantify total cell proliferation prior to and following replating, EdU incorporation assay was used to detect all cells that were in the S phase of cell cycle within 24-h intervals (Figure 2(a)). GFP^+^ cells were identified by high ratio of GFP to RFP (Tomato) signal, while EdU^+^ cells were identified based on the fluorescence integrated over the whole cell nucleus (Figure 2(b)). GFP^−^ cells displayed high incidence of DNA replication throughout the seven-day time course, with a prominent increase between 24 and 48 h after replating. In comparison, GFP^+^ cells displayed a much lower propensity for EdU incorporation, irrespective of TGF-β treatment (Figure 2(c)). In conclusion, non-adipocyte cells present in the SVF of mouse adipose tissue display prolonged proliferation *in vitro*, in contrast to primary adipocytes.

(a) Schematic of the experiment to quantify cells in the S/G2 phases of cell cycle in SVF-derived adipocytes using EdU incorporation assay over 6 d after replating. EdU was added to culture media for 24 h prior to fixation and staining. (b) A representative density plot of single-cell nGFP/nTomato signal measured over the nucleus vs. integrated nuclear EdU signal. Dotted lines represent cut-offs used to gate for GFP^+^ and EdU^+^ cells. (c) Quantification of EdU^+^ cells within GFP^+^ and GFP^−^ populations of differentiated SVF cells over 6 d after replating. *n* = 4-6 replicates, average ± S.E.M. is shown.

Even though the ceiling culture method depends on low density of lipid-laden adipocytes, contamination by non-adipocytes seems likely. Although adipose tissue volume comprises mainly of adipocytes, single-cell transcriptome studies show that they amount to less than 20% of all cells in the tissue, in addition to endothelial cells, mesenchymal stem cells, preadipocytes and blood cells [2].

## Discussion

True adipocyte dedifferentiation *in vivo*, which includes adipocytes undergoing not only morphological changes, but also cell cycle re-entry, has been reported in the context of mammary gland plasticity related to lactation and weaning [5], and dermal adipose tissue plasticity associated with hair cycling and skin wound healing [6] (Table 1). However, in both these contexts, the dedifferentiated cells underwent re-differentiation exclusively into adipocytes. It is unknown whether dedifferentiated adipocytes *in vivo* are unipotent or whether the microenvironmental cues control their re-differentiation into adipocytes. This contrasts with the reported DFAT cells, which are multipotent. Based on our experimental results, we conclude that this discrepancy in the differentiation ability can be attributed to the contamination of adipocytes in the ceiling culture by adjacent non-adipocyte cells, most likely MPPs, which are known to be both highly proliferative and capable of multilineage differentiation. Previously, a similar lineage tracing approach derived DFAT cells from adipose tissue of Fabp4-Cre/LacZ-ROSA (R26R) mice and showed the derivation of multipotent colonies with positive β-gal activity [15]. However, it has been shown that *Fabp4* (*aP2*) expression in adipose tissue is not restricted to adipocytes [11], which limits the usability of *Fabp4* as an adipocyte lineage marker and likely explains the discrepancy between our results and the previous report.

**Table 1.** Studies that investigated adipocyte cell cycle re-entry and proliferation in mice *in vivo* using lineage tracing methods.

Tissue	Context	Finding	Adipocyte tracing model	Reference
Mammary gland adipose tissue	Mammary gland remodelling related to lactation	BrdU incorporation observed in cells arising from lineage-traced adipocytes after weaning	Adipoq:rtTA TRE:Cre mT/mG (AdipoChaser mT/mG)	Wang et al. [5]
Dermal adipose tissue	Changes in dermal adipose tissue associated with wound healing, hair cycling	Dedifferentiation and redifferentiation of dermal adipocytes under physiological and pathophysiological conditions; BrdU incorporation detected in dedifferentiated adipocytes	AdipoChaser mT/mG	Zhang et al. [6]
Inguinal adipose tissue transplanted into skin lesions	Fat grafting as a method to reverse skin fibrosis in a scleroderma model	Dedifferentiation and re-differentiation of adipocytes after fat grafting; proliferation or cell cycle re-entry not studied	Adipoq:CreER mT/mG	Wang et al. [17]

Due to the technical limitations, it is impossible to rule out the existence of differences in the molecular mechanisms of human and mouse adipocyte dedifferentiation. Therefore, to test the cell of origin of human DFAT cells, in the future experimental approaches that use lineage-tracing approaches could be incorporated into human DFAT isolation protocols. For example, lentiviral vectors, which are capable of infecting nondividing cells, could be used to deliver genetic constructs to primary human adipocytes.

In summary, we conclude that DFAT cells are likely derived from non-adipocyte populations present in adipose tissue. Concerns regarding the purity of adipocytes isolated by the commonly used ceiling culture method have been raised previously [16]. Due to the long time of the procedure, a minuscule population of highly proliferative cell population, such as MPPs, is likely to overgrow the culture.

Further optimization of the DFAT isolation protocol could improve the purity and/or survival of the isolated population and increase the percentage of GFP^+^ cells present in the sample. Additional steps, such as purification of GFP^+^ cells using fluorescence-activated cell sorting and increasing the EdU treatment window, could also allow for better detection of rare proliferative adipocyte-derived cell populations. Given that cell cycle re-entry was observed in adipocyte-derived cells *in vivo* [5,6], it is possible that the time frame of 30 d did not allow for cell cycle re-entry of adipocytes, and increasing the time between cell isolation to cell cycle assessment would be required to observe cell cycle re-entry in adipocytes that were dedifferentiated *ex vivo*.

## Data Availability

The data that support the findings of this study are available on Zenodo (doi: 10.5281/zenodo.11193934).
